# Effects of urinary extracellular vesicles from prostate cancer patients on the transcriptomes of cancer-associated and normal fibroblasts

**DOI:** 10.1186/s12885-022-10107-3

**Published:** 2022-10-12

**Authors:** Lilite Sadovska, Pawel Zayakin, Cristina Bajo-Santos, Edgars Endzeliņš, Jānis Auders, Laura Keiša, Juris Jansons, Vilnis Lietuvietis, Aija Linē

**Affiliations:** 1 Latvian Biomedical Research and Study Centre, Ratsupites Str 1, k-1, LV-1067 Riga, Latvia; 2 Faculty of Medicine, University of Latvia, Raina blvd. 19, 1586 LV Riga, Latvia; 3 Riga Stradiņš University, Dzirciema Str 16, LV-1007 Riga, Latvia

**Keywords:** Extracellular vesicles, Prostate cancer, RNA sequencing, Transcriptome, cancer-associated fibroblasts, Biomarker

## Abstract

**Background:**

Increasing evidence suggests that cancer-derived extracellular vesicles (EVs) alter the phenotype and functions of fibroblasts and trigger the reprogramming of normal fibroblasts into cancer-associated fibroblasts (CAFs). Here, we for the first time studied the effects of urinary EVs from PC patients and healthy males on the transcriptional landscape of prostate CAFs and normal foreskin fibroblasts.

**Methods:**

Patient-derived prostate fibroblast primary cultures PCF-54 and PCF-55 were established from two specimens of PC tissues. EVs were isolated from urine samples of 3 patients with PC and 2 healthy males and used for the treatment of prostate fibroblast primary cultures and normal foreskin fibroblasts. The EV-treated fibroblasts were subjected to RNA sequencing analysis.

**Results:**

RNA sequencing analysis showed that the fibroblast cultures differed significantly in their response to urinary EVs. The transcriptional response of foreskin fibroblasts to the urinary EVs isolated from PC patients and healthy controls was very similar and mostly related to the normal functions of fibroblasts. On the contrary, PCF-54 cells responded very differently - EVs from PC patients elicited transcriptional changes related to the regulation of the cell division and chromosome segregation, whereas EVs from healthy males affected mitochondrial respiration. In PCF-55 cells, EVs from both, PC-patients and controls induced the expression of a number of chemokines such as CCL2, CCL13, CXCL1, CXCL8, whereas pathways related to regulation of apoptotic signaling and production of cell adhesion molecules were triggered specifically by EVs from PC patients.

**Conclusion:**

This study demonstrates that urinary EVs from PC patients and healthy controls elicit distinct transcriptional responses in prostate CAFs and supports the idea that EVs contribute to the generation of functional heterogeneity of CAFs. Moreover, this study suggests that the changes in the gene expression pattern in EV recipient cells might serve as a novel type of functional cancer biomarkers.

**Supplementary Information:**

The online version contains supplementary material available at 10.1186/s12885-022-10107-3.

## Background

Cancers are highly complex ecosystems consisting of cancer cells, extracellular matrix, and stromal cells. Cancer-associated fibroblasts (CAFs) are the major cellular stromal component in various solid tumors, including prostate cancer (PC). A substantial fraction of CAFs are derived from resident fibroblasts, however, they also have been shown to originate from other mesoderm-derived cell types [1]. They are characterized by the expression of a set of relatively selective but non-specific markers, including vimentin, FAP, PDGFRB, FSP1, and α-SMA, and share a high degree of similarity with myofibroblasts [2]. Prostate CAFs are heterogeneous and dynamic cells that play diverse roles in the progression of PC, acquisition of drug resistance, and metastasis [2]. Single-cell RNA sequencing has revealed CAF subpopulations that differ in the expression of chemotactic chemokines and may have unique functions within the tumor microenvironment (TME), including a role in immune and inflammatory cell recruitment [3]. Conceivably, the heterogeneity and plasticity of CAFs is driven by the signals they receive from cancer and stromal cells in the TME.

Extracellular vesicles (EVs) have emerged as very important mediators of crosstalk between tumor and stromal cells. The term “EV” refers to all types of plasma membrane-delimited particles that are naturally released from the cell and cannot replicate, and include various subtypes that differ in their biogenesis, size and physical properties, molecular composition, and functions in the body [4, 5]. EVs are released by virtually all cell types in the body, and they have been found in various body fluids, including blood, urine, semen, milk, saliva, etc. [5–7]. EVs transfer proteins, lipids, metabolites, mRNAs, and various non-coding RNAs, and even DNA fragments between cells [5]. EVs can be internalized by the recipient cells and trigger various intracellular signal transduction pathways [5, 8] or bind to the cell surface receptors and trigger the respective downstream signaling pathway [9, 10].

A growing body of evidence suggests that cancer-derived EVs promote cancer progression by transferring aggressive phenotypic traits to other cancer cells, modulating the anti-tumor immune response, remodeling the TME, and promoting the formation of pre-metastatic niche [11]. Exposure of fibroblasts to cancer-derived EVs has been shown to promote the acquisition of CAF phenotype [12, 13] and induce the secretion of various chemokines [14], or even induce transformation of repair-defective fibroblasts into cancer cells [15]. Moreover, the secretome of EV-activated fibroblasts, composed of soluble and EV-associated molecules, was shown to modulate the properties of fibroblasts, tumor and endothelial cells thus promoting the tumor progression [16]. Urine is a rich source of cancer-derived EVs in PC patients [17, 18]. We hypothesized that urinary EVs from PC patients may affect the phenotype and functions of fibroblasts in a similar manner than PC-derived EVs activate the fibroblasts locally in the TME. Thus, the analysis of their effects on fibroblasts in vitro may inform about the ways how patient’s tumor shapes the TME, serving as a functional biomarker that provides important information about the presence and behavior of cancer. The goal of the current study was to gain an insight into the effects of urinary EVs from PC patients and healthy males on the transcriptional landscape of prostate CAFs and cancer-naïve foreskin fibroblasts.

## Methods

### Clinical samples

Prostate cancer specimens and urine samples were collected from PC patients diagnosed and treated at Riga East University Hospital. Fresh prostate cancer tissue specimens were obtained on the day of surgery by an experienced pathologist, placed into RPMI-1640 medium with 1x Antibiotic-Antimycotic (Sigma-Aldrich, USA). Whole morning urine samples from PC patients were collected before any manipulations and medication on a day before surgery. The samples were centrifuged for 15 min at 2000* g* to remove cellular debris, aliquoted, and stored at -80 °C until analysis. In addition, morning urine samples from age-matched healthy men were obtained from the Latvian Genome Database.

The study was conducted according to the Declaration of Helsinki. The specimens were collected after the patients’ informed written consent was obtained and the research on anonymized patients’ samples was approved by the Latvian Central Medical Ethics Committee (decision No. 01-29.1/488).

### Cell culture

Prostate fibroblast primary cultures were obtained from the surgical specimens as described by Navone et al. [19]. Briefly, the tumor was minced into small pieces and grown in RPMI-1640 (Invitrogen, USA) complete medium supplemented with 10% FBS (Sigma-Aldrich, USA), 2mM L-glutamine (Sigma-Aldrich, USA), 100 units/ml primocin (Sigma-Aldrich, USA) in a humidified 5% CO_2_ atmosphere at + 37˚C. When the cells from the tissue samples formed a monolayer, fibroblasts were separated by differential trypsinization, where primary tissue culture was first trypsinized with 0.25% trypsin for 3 min and this fraction was seeded and grown further as fibroblasts, and the epithelial cells were trypsinized for 7 more minutes. Fibroblasts were grown in DMEM-F12 complete medium supplemented with 10% FBS (Sigma-Aldrich, USA), 2mM L-glutamine (Sigma-Aldrich, USA), 100 units/ml primocin (Sigma-Aldrich, USA) and 10 ng/ml fibroblast growth factor (FGF) (Sigma-Aldrich, USA) in a humidified 5% CO_2_ atmosphere at + 37˚C.

The human dermal fibroblast line Hs68 was obtained from ATCC (Manassas, VA, USA). The Hs68 cells were cultured in DMEM-F12 (Invitrogen, USA), supplemented with 2mM L-glutamine (Sigma-Aldrich, USA), 10% fetal calf serum (FCS) (Sigma-Aldrich, USA), 1x Antibiotic-Antimycotic and 100 units/ml primocin (Sigma-Aldrich, USA), in a humidified 5% CO_2_ atmosphere at + 37˚C.

For EV uptake and RNA sequencing analysis, the cells were grown with EV-depleted FBS, prepared by ultracentrifugation for 1.5 h at 100,000* g* + 4 °C.

### Immunofluorescence

Hs68, PCF54 and PCF55 cells were seeded in DMEM-F12 complete medium at a density of 1 × 10^4^ cells per well in 24-well plates on glass coverslips and grown as a monolayer for 24 h. After that, the cells were rinsed with PBS, fixed, and permeabilized with methanol-acetone (1:1) in -20 °C for 20 min, then washed with PBS and blocked with 2% BSA. Cells were incubated with αSMA primary antibody (sc-32,251, Santa Cruz Biotechnology, USA; 1:50 dilution in PBS, 1% BSA) overnight in + 4 °C and with Cy3-anti mouse secondary antibody (115-165-071, Jackson Immunoresearch, UK) for 1 h in room temperature in dark. Next, cells were washed with PBS and mounted on glass slides in ProLong™ Gold Antifade Mountant with DAPI (Thermo Fisher Scientific, USA) and incubated in + 4˚C, overnight. Fluorescence imaging was done on Leica DM3000 microscope (Leica Microsystems GmbH, Germany).

### Isolation of extracellular vesicles

EVs were isolated from the urine samples using size exclusion chromatography (SEC) as described before [20] with some modifications. Briefly, the urine samples were thawed at + 37˚C in a water bath and centrifuged at 10,000* g* for 15 min at + 4˚C to remove uromodulin. Next, the samples were concentrated up to 1 ml using 100 kDa centrifugal filters (Merck Millipore, USA) and fractionated on Sepharose CL2B 10 ml columns. The eluate was collected in 12 sequential 0.5 ml fractions and each fraction was measured with Zetasizer Nano ZS (Malvern, UK). Fractions containing particles larger than 30 nm were combined and concentrated up to 100 µl using 3 kDa centrifugal filters (Merck Millipore, USA). EVs were aliquoted and stored at -80˚C until use. The purity, size distribution profile and concentration of EVs were assessed by transmission electron microscopy (TEM) and nanoparticle tracking analysis (NTA) using NanoSight NS300 instrument (Malvern, UK). For TEM, EV samples were put on 300-mesh carbon coated EM grid, incubated for 5 min and negatively stained with 1% uranyl formate (*w/v*) for 1 min and dried. The microscopy was done with JEM-1230 transmission electron microscope (JEOL, USA). For NTA, EV samples were diluted in 0.02 μm filtered PBS, and for each sample five 60 s videos were recorded, and data analysis was performed with NanoSight NTA Software in the auto mode.

### Western blot

Urinary EVs were heated for 5 min at 95˚aC with reducing Laemmli buffer and amounts corresponding to 1.5ml urine were loaded per each lane of a 10% SDS-PAGE gel. LNCaP cell lysate, prepared in 1x RIPA and heated with reducing Laemmli prior to loading, was used as a control (10 µg proteins per lane). PageRuler™ Prestained Protein Ladder, 10 to 180 kDa (ThermoFisher Scientific) was loaded onto each gel for assessment of protein molecular weighs. After separation by SDS-PAGE, the proteins were transferred to Amersham Protran Supported 0.45 NC membranes (Merck Millipore), which were subsequently blocked using 10% (w/v) low-fat milk. Membranes were incubated with primary antibodies against TSG101 (Abcam, #ab15011, 1:1000 dilution), Calnexin (Abcam, #ab22595, 1:2000 dilution), or CD63 (Sino Biological, # 11271-T16, 1:300 dilution) overnight at + 4 ˚C. Membranes were washed in TBST and incubated for 1 h at room temperature with anti-rabbit IgG, F(ab’)2-HRP (Santa Cruz, #sc-3837, 1:2000 dilution), or goat anti-mouse m-IgG BP-HRP (Santa Cruz, # sc-516,102, 1:2000 dilution). After washing again in TBST, immunoreactive bands were visualized using Western Blotting Detection Reagent kit (GE HealthCare), and pictures were taken using a Nikon d610 dSLR body (Nikon) with Sigma 35 mm f/1.4 DG HSM Art lens (Sigma).

### EV uptake

Isolated EVs (1 × 10^8^ EVs per sample, calculated based on NTA data) were labeled with PKH67 green membrane dye (Sigma Aldrich, USA) according to the manufacturer’s protocol. Briefly, the EVs were diluted in Diluent C and stained with 0.5 µl of PKH67 for 5 min at room temperature. After that, 100 µl of 1% BSA was added and the excess dye was removed with Invitrogen™ Exosome Spin Columns (MW 3000) (Invitrogen, USA).

Hs68, PCF54 and PCF55 cells were seeded in DMEM-F12 complete medium at a density of 1 × 10^4^ cells per well in 24-well plates on glass coverslips and grown as a monolayer for 24 h. PKH67-labelled EVs (1 × 10^4^ EVs/cell) were added to the cells and incubated for 1 h, 2 h, 4 h, 15 h, 24 h, or 48 h in a humidified 5% CO_2_ atmosphere at 37˚ C. At these time points, the cells were fixed with 4% formaldehyde for 10 min in + 37˚C, washed with PBS, and mounted on glass slides in ProLong™ Gold Antifade Mountant with DAPI (Thermo Fisher Scientific, USA). Confocal fluorescence imaging was done on Leica TCS SP8 confocal laser scanning microscope (Leica Microsystems GmbH, Germany), fluorescence imaging was done on Leica DM3000 microscope (Leica Microsystems GmbH, Germany).

### Treatment of cells with EVs and RNA extraction

Hs68, PCF-54 and PCF-55 cells were seeded in DMEM-F12 complete medium at a density of 1 × 10^5^ cells per well in 12-well plates and 1 × 10^4^ EVs per cell (total 1 × 10^9^ EVs/well) were added to the cells and they were grown as a monolayer for 48 h. Untreated control cells were grown in parallel. After that, the cells were washed with PBS and lysed with 1ml TRI-Reagent® (Sigma Aldrich, USA) and RNA was extracted according to the manufacturer’s protocol. Briefly, 200 µl of chloroform was added, the samples were centrifuged at 12,000* g* for 15 min at + 4 °C, the aqueous phase was transferred to a new tube and isopropanol was used to precipitate the RNA by centrifuging at 12,000* g* for 10 min at + 4˚C. After that, the RNA was washed with 70% Ethanol-DEPC and the DNA was removed with DNA-free™ (Thermo Fisher Scientific, USA) DNA removal kit according to the manufacturer’s protocol. The RNA concentration was measured with NanoDrop 1000 (Thermo Ficher Scientific, USA) and the integrity of the RNA was measured with Agilent Bioanalyzer using RNA Pico chip (Agilent Technologies, USA).

### RNA sequencing and data analysis

For transcriptome sequencing, the rRNA was removed from 200 ng of total RNA using MGIEasy rRNA depletion kit (MGI, China). The transcriptome libraries were built using MGIEasy RNA Directional Library Prep Kit (MGI, China) according to the manufacturer’s protocol. Briefly, the RNA was fragmented into 250 bp pieces, and it was reverse transcribed, and the second strand was synthesized. Adapters were ligated according to the manufacturer’s recommendations and the product was amplified with PCR. The library insert length was measured with Agilent Bioanalyzer using High Sensitivity DNA chip (Agilent Biotechnologies, USA) and the concentration of the libraries was measured using Qubit® fluorometer (Thermo Fisher Scientific, USA). Libraries from untreated control cells were constructed in duplicates (starting from cells grown in different wells). The libraries were pooled for circularization accordingly to the manufacturer’s recommendations and the libraries were sequenced with MGI DNBSEQ-G400 sequencer.

The obtained raw data in fastq format were analyzed using ad-hoc R script pipeline, which included the trimming of adapters using cutadapt [21], mapping of the reads using STAR [22] against Ensembl human genome (GRCh38), allowing only unique alignments to be counted using Rsubread package [23] with Ensembl human genome annotation (GRCh38.p13). For differentially expressed gene (DEG) analysis, the reads were normalized and analyzed using DESeq2 [24] package. A subset of DEGs (adj. P < 0.05 and abs(logFC) > 0.5) was subjected to GO term analysis using GOstats [25] and enrichment analyses using rentrez package [26], GO.db package [27], and ShinyGO package [28].

## Results

### Isolation and characterization of urinary EVs

**Fig. 1:**
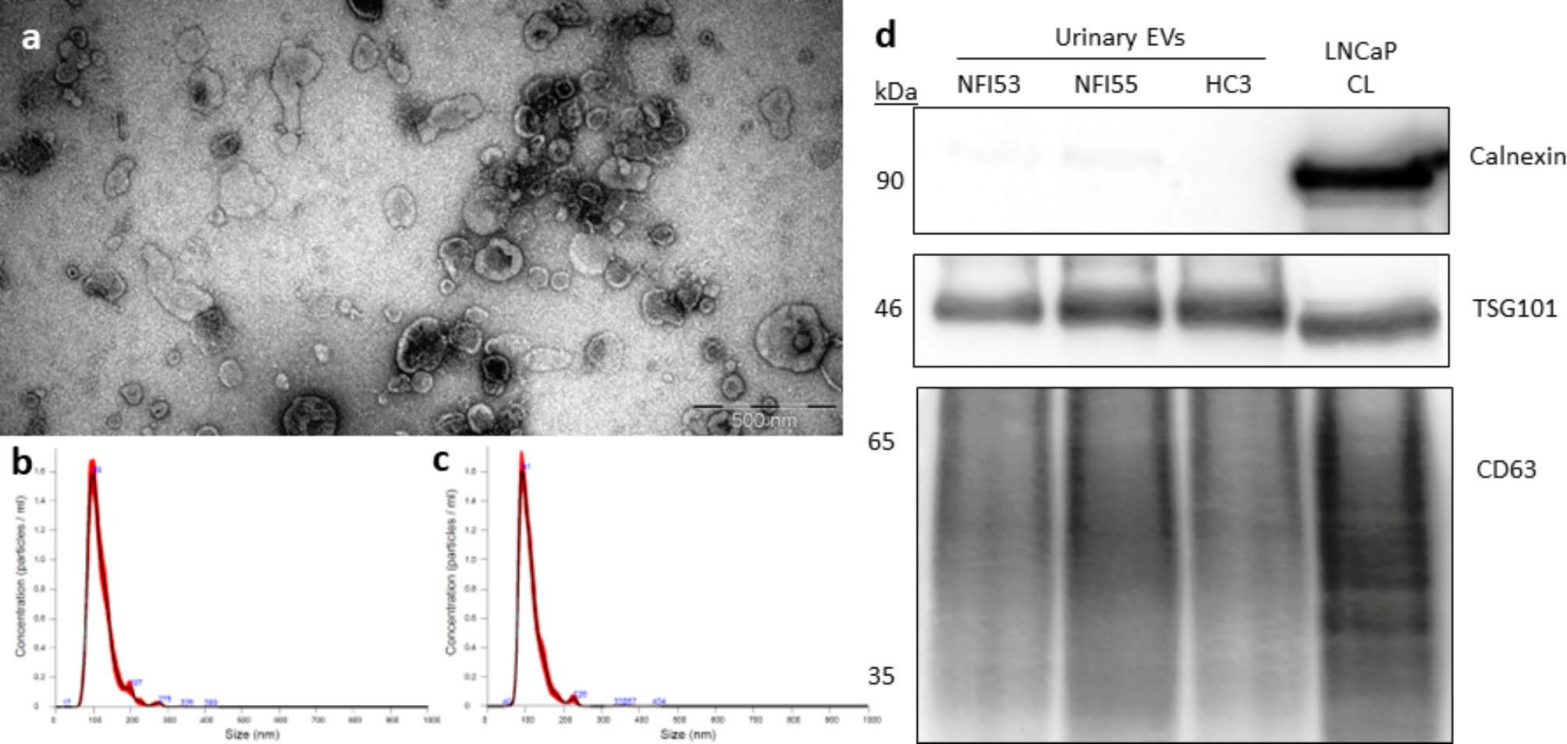
The yield and size of urinary EVs from PC patients and healthy controls. **a**, Representative transmission electron microscopy image of urinary EVs from a PC patient. **b-c**, Nanoparticle tracking analysis of urinary EVs from a patient with PC and healthy male, respectively. **d**, Western blot analysis of EV markers (TSG101 and CD63) and an endoplasmic reticulum protein Calnexin (negative control) in urinary EVs from 2 PC patients (NFI53, NFI55) and a healthy control (HC3), and LNCaP cell lysate (CL).

EVs were isolated from urine samples (15 ml) of 3 PC patients, including those two patients whose tumor tissues were used for establishing PCF-54 and PCF-55 fibroblast lines, and 2 healthy males (HC). All patients had stage pT2N0M0, moderately differentiated PC with Gleason score of 7 (3 + 4). The yield, size, and purity of EVs were assessed by TEM and NTA. TEM images revealed that the EV samples contain vesicles of various sizes, from 50 to 200 nm, most having a cup-shape morphology typically observed for exosomes by TEM (Fig. 1a). NTA showed that the EV concentration ranged from 6.6 × 10^7^ to 1.9 × 10^8^ particles per ml of urine and the majority of the particles were in the size range from 80 to 330 nm (Fig. 1b,c). We did not observe significant differences in the size or concentration of EVs from PC patients and healthy controls. Western blot analysis showed that the EV samples were positive for typical EV markers CD63 and TSG101, but negative for calnexin, an endoplasmic reticulum protein, thus showing that the EV preparations do not contain significant contamination of ER membranes (Fig. 1d; additional files 1,2,3).

### Generation of patient-derived prostate fibroblast primary cultures

**Fig. 2:**
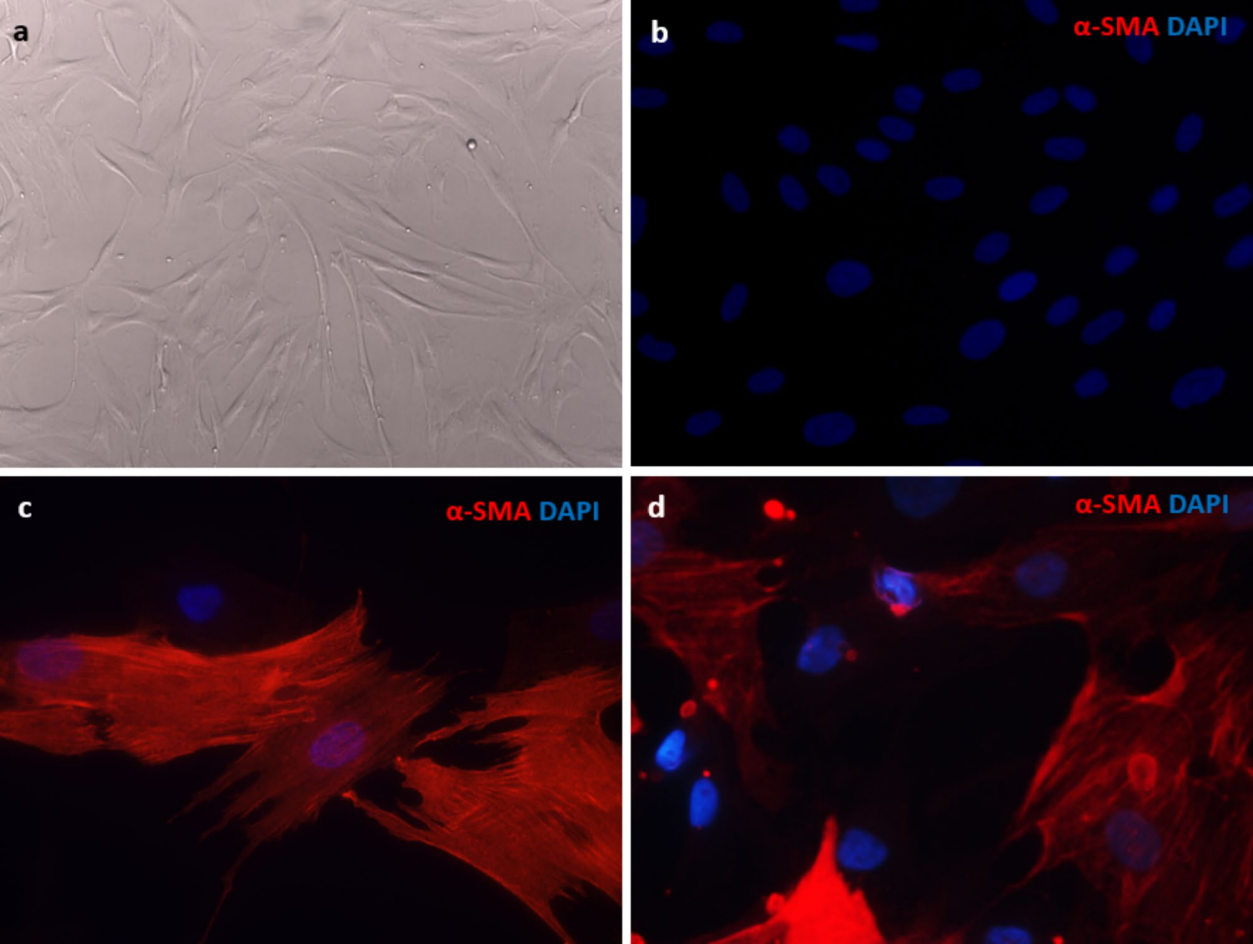
Microscopy pictures of fibroblasts. **a**, brightfield image of PCF-54 fibroblasts after separation from other primary cells, 20x magnification. **b-d**, αSMA expression in Hs68 (b), PCF-54 (c) and PCF-55 (d) fibroblasts, fluorescence microscopy, 63x magnification

Patient-derived prostate fibroblast primary cultures were generated from surgical specimens of two PC patients and were designated as PCF-54 and PCF-55. These cells exhibited spindle-like morphology that is typical for fibroblasts (Fig. 2a). In this experiment, eight passage of these cells was used.

The fibroblast cultures were characterized by immunofluorescence staining for αSMA expression, one of the most common CAF markers [2]. Both, PCF-54 and PCF-55 cells were positive for αSMA, however, to varying degrees: approx. 70% of the PCF-54 cells and 50% of the PCF-55 cells were positive for αSMA (Fig. 2b-d). This suggests that both fibroblast cultures contain activated CAFs. As it could be expected, foreskin fibroblasts Hs68 were negative for αSMA.

### Time-course of EV uptake by dermal fibroblasts

**Fig. 3:**
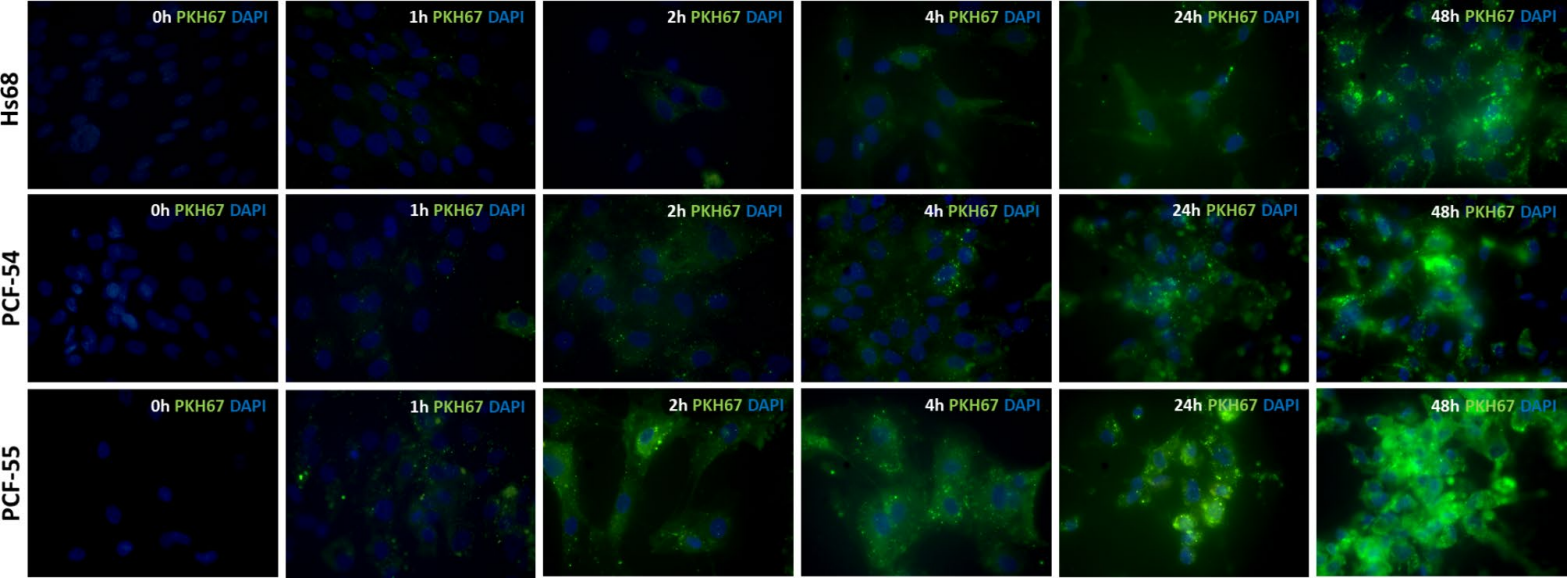
Uptake of urinary EVs by Hs68, PCF-54 and PCF-55 cells. Urinary EVs from a PC patient were labelled with PKH67 and their uptake by Hs68, PCF-54 and PCF-55 cells was studied by fluorescence microscopy, 63x magnification

The time course of EV uptake by Hs68, PCF-54 and PCF-55 cells was analyzed with fluorescence microscopy, using PKH67 labeled urinary EVs from the patient PC-55 (Fig. 3). After the first hour, the EVs are visualized as separate fluorescent dots, but later the fluorescence was spread in the recipient cells, and EVs accumulated in the recipient cells in the subsequent time points. The uptake rate in Hs68 cells appeared somewhat slower than in PCF-54 and PCF-55 cells during the first 24 h, however at 48 the differences were leveled up, suggesting that after 48 h most of the EVs have been internalized and/or bound to the recipient cell surface.

### Transcriptional response of prostate cancer-associated fibroblasts and dermal fibroblasts to urinary EVs

To assess the impact of urinary EVs from PC patients on the transcriptome of cancer-naïve foreskin fibroblasts and prostate cancer-associated fibroblast primary cultures, Hs68, PCF-54, and PCF-55 cells were exposed to EVs isolated from urine samples of 3 PC patients and 2 healthy controls and subjected to RNA sequencing analysis. Among the PC patients were PC-54 and PC-55 –the patients whose tumor tissues were used for establishing PCF-54 and PCF-55 fibroblast lines. On average, 29 million raw reads were obtained for each library. The reads were mapped against Ensembl human genome (GRCh38). On average, 91% of the reads were uniquely mapped and retained for further analysis, and approximately 6% of reads per library were multi-mapped and excluded from further analysis.

**Fig. 4:**
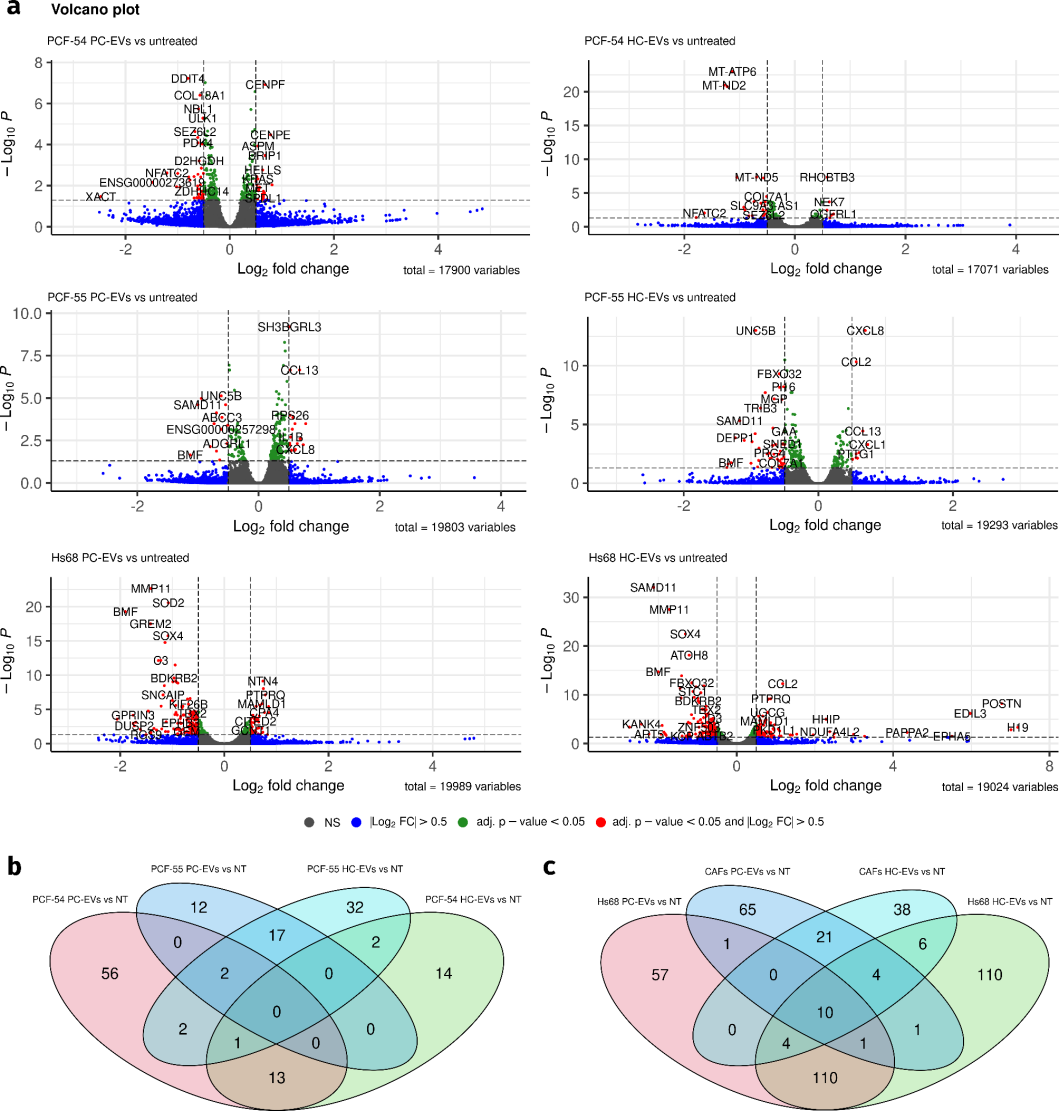
Transcriptional alterations in fibroblasts treated with urinary EVs. **a**, Volcano plots depicting DEGs in PCF-54, PCF-55 and Hs68 cells treated with urinary EVs from PC patients (PC-EVs) and healthy controls (HC-EVs) as compared to the untreated cells. **b**, Venn diagram showing the number and overlap of DEGs in PCF-54 and PCF-55 cells treated with PC-EVs and HC-EVs. **c**, Venn diagram showing the number and overlap of DEGs in Hs68 and merged PCF-54 and PCF-55 cells (CAFs).

To identify differentially expressed genes (DEGs) that are regulated by urinary EVs, the fibroblast samples treated with EVs from PC patients (PC-EVs) or healthy controls (HC-EVs) were compared to the untreated cells using DESeq2 tool. Only protein-coding genes and long non-coding RNAs were included in the differential expression analysis. Both, PC-EVs and HC-EVs elicited substantial changes in the gene expression profile of all three fibroblast lines (Fig. 4a). In PCF-54 cells, 75 and 30 DEGs regulated by PC-EVs and HC-EVs, respectively, were found and 14 of them were overlapping, while in PCF-55 cells, 31 and 56 DEGs including 19 overlapping DEGs were found (LogFC > 0.5 and adj. *P* < 0.05). Only 2 DEGs regulated by PC-EVs and 3 DEGs regulated by HC-EVs were common between PCF-54 and PCF-55 cells (Fig. 4b). The number of genes regulated by autologous EVs was not substantially different from that induced by allogeneic EVs in both cell lines. In Hs68 cells, 183 and 246 transcripts were altered by PC-EVs and HC-EVs, respectively and 125 of them were overlapping (LogFC > 0.5 and adj. *P* < 0.05). Only 12 of the PC-EV-regulated genes and 24 HC-EV regulated genes were common between Hs68 cells and cancer-associated fibroblasts (merged PCF-54 and PCF-55) (Fig. 4c). A full list of DEGs is provided in Additional file 4.

We did not observe significant alterations in the expression level of common CAF markers including α-SMA, FAP, PDGFRB, FSP1, and vimentin in Hs68 and PCF-54 cells treated with urinary EVs from PC patients and healthy males. In PCF-55 cells, a moderate decrease in the expression level of PDGFRB was found both in HC-EV-treated cells (LogFC=-0.34; adj. *P* = 0.0004) and PC-EV-treated cells (LogFC=-0.24; adj. *P* = 0.02). whereas the other CAF markers were not altered.

### Biological processes affected by urinary EVs

**Fig. 5:**
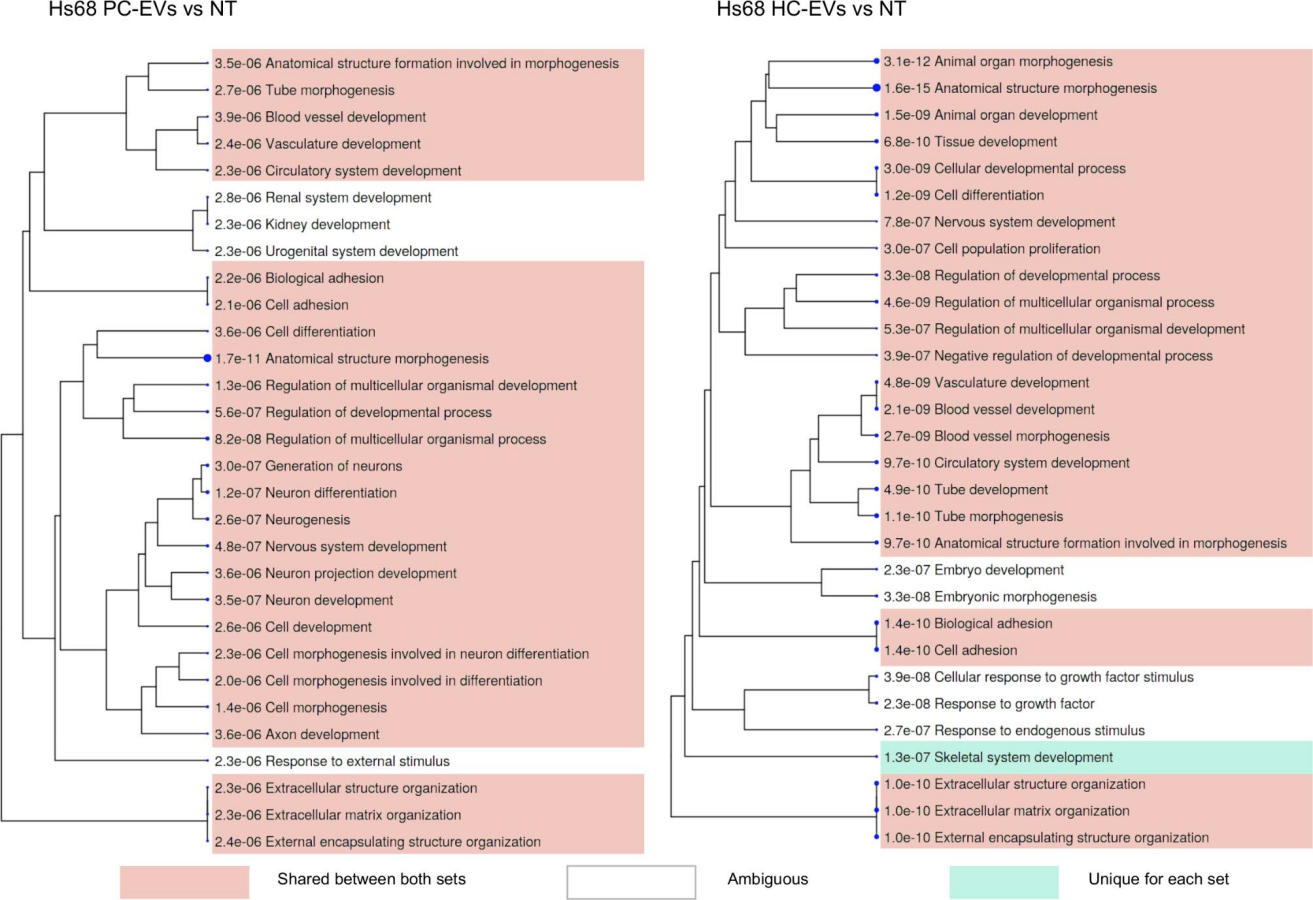
GO term enrichment analysis in Hs68 cells treated with PC-EVs and HC-EVs. A hierarchical clustering tree summarizing the correlation among significant pathways represented by DEGs in Hs68 cells treated with PC-EVs or HC-EVs vs. untreated cells. Pathways with many shared genes are clustered together. Bigger dots indicate more significant P-values. Pathways that are shared by HC-EV and PC-EV treated cells are highlighted in green, pathways that are unique for either HC-EV or PC-EV treated cells are highlighted in pink, pathways that are enriched in similar yet not identical gene sets are left blank. The top 30 pathways with adj. *P* < 0.05 are shown

To explore the biological significance of DEGs, GO term enrichment and clustering analysis were carried out. The analysis revealed a significant number of biological processes and pathways regulated by PC-EVs and HC-EVs in all cell lines studied. In Hs68 cells, the most significantly enriched pathways were related to normal functions of fibroblasts - extracellular matrix organization, cell and organ morphogenesis and various developmental processes - and were shared between the cells treated with PC-EVs and HC-EVs (Fig. 5).

**Fig. 6:**
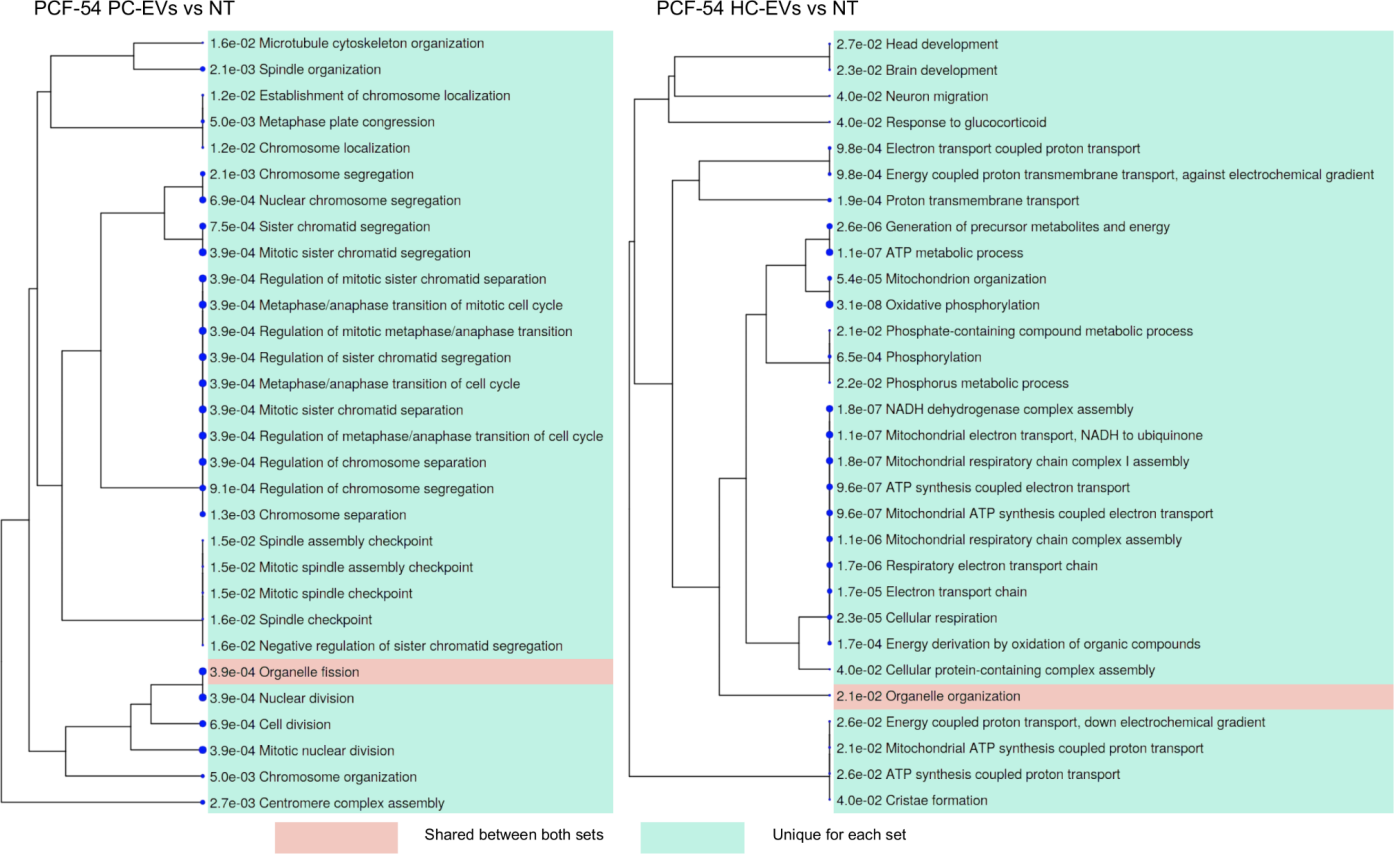
GO term enrichment analysis in PCF-54 cells treated with PC-EVs and HC-EVs. A hierarchical clustering tree summarizing the correlation among significant pathways represented by DEGs in PCF-54 cells treated with PC-EVs or HC-EVs vs. untreated cells. Pathways with many shared genes are clustered together. Bigger dots indicate more significant P-values. Pathways that are shared by HC-EV and PC-EV treated cells are highlighted in green, pathways that are unique for either HC-EV or PC-EV treated cells are highlighted in pink. The top 30 pathways with adj. *P* < 0.05 are shown

On the contrary, PCF-54 cells responded very differently to the PC-EV and HC-EV signaling. The majority of the processes affected by HC-EVs were related to cellular energetics, oxidative phosphorylation, and mitochondrial electron transport (represented by downregulation of *ATP6, MT-ND2, MT-ND1, MT-ND5, MT-ND6, DDIT4, MT-ND3, MT-ND4, ATP8* etc.), various tissue developmental processes (downregulation of *COL18A1,COL7A1, BGN, NTN1* and *ADAMTSL4* and upregulation of *CENPF* and *KRAS*) and response to glucocorticoid signaling (downregulation of *DDIT4/REDD1* and *MT-ND3* and upregulation of *KRAS*). Whereas PC-EVs affected entirely different processes: chromosome segregation, metaphase/anaphase transition and mitotic spindle organization, nuclear and cell division (upregulation of *CENPF, CENPE, ASPM, BRIP1, HELLS, RB1, CCNB2, KNTC1, USP16, SPDL1, NCAPG etc.*) (Fig. 6).

**Fig. 7:**
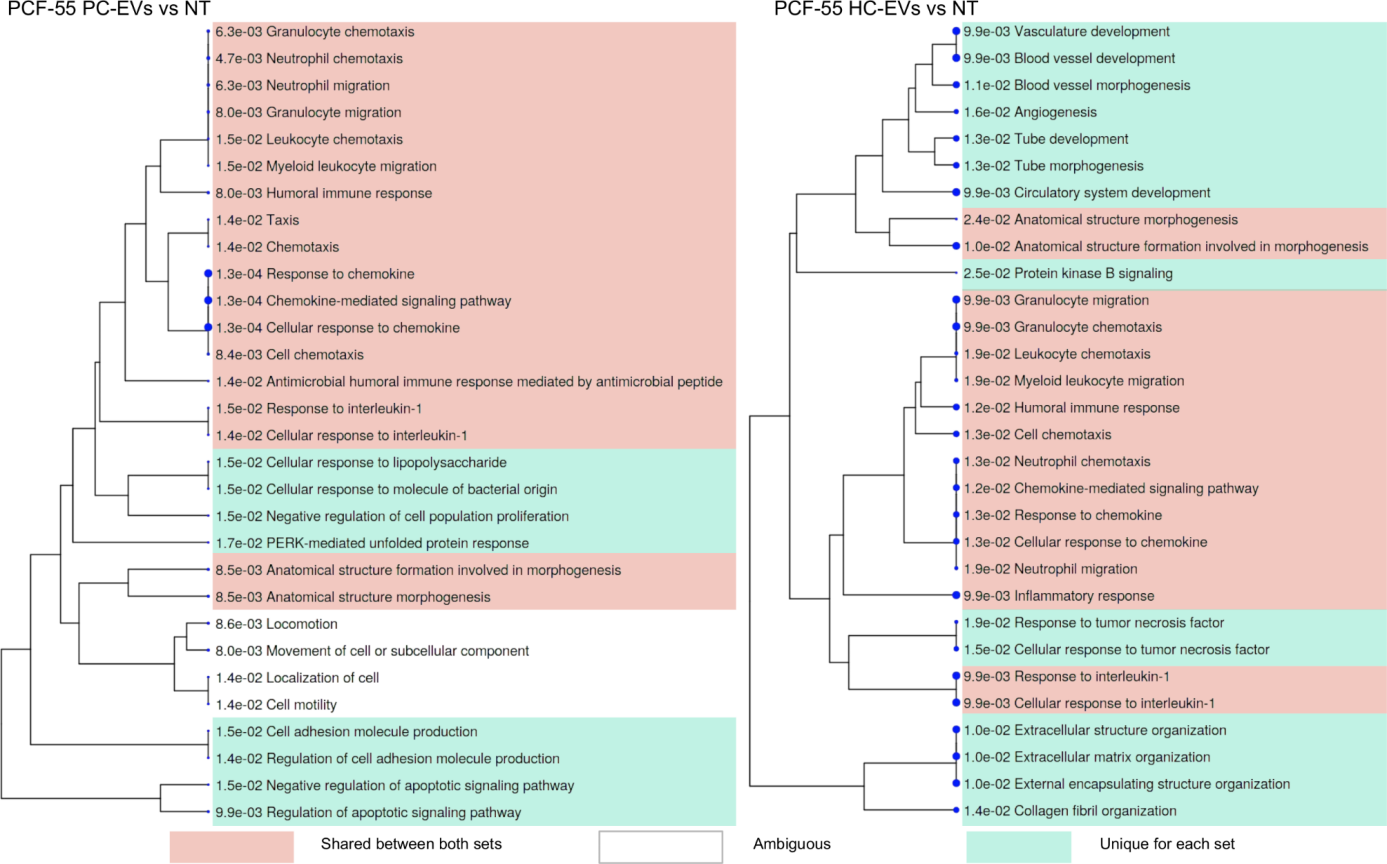
GO term enrichment analysis in PCF-55 cells treated with PC-EVs and HC-EVs. A hierarchical clustering tree summarizing the correlation among significant pathways represented by DEGs in PCF-55 cells treated with PC-EVs or HC-EVs vs. untreated cells. Pathways with many shared genes are clustered together. Bigger dots indicate more significant P-values. Pathways that are shared by HC-EV and PC-EV treated cells are highlighted in green, pathways that are unique for either HC-EV or PC-EV treated cells are highlighted in pink, pathways that are enriched in similar yet not identical gene sets are left blank. The top 30 pathways with adj. *P* < 0.05 are shown

In PCF-55 cells, both PC-EVs and HC-EVs stimulated the expression of various chemokines and cytokines (such as CXCL8, CCL2, CCL13, CXCL1, IL1B etc.) that are induced in response to interleukin-1 and regulate cell chemotaxis and migration of various immune cells. However, the regulation of apoptotic signaling, cell adhesion molecule production and cellular response to molecules of bacterial origin were affected specifically by PC-EVs (Fig. 7).

## Discussion

EVs released by cancer cells have been shown to be internalized by fibroblasts in vitro and in vivo [12, 29]. Increasing evidence suggests that cancer-derived EVs affect the phenotype and functions of fibroblasts and facilitate their reprogramming into CAFs. For example, melanoma-derived EVs stimulated the expression of α-SMA and FAP in embryonic fibroblasts [30]. Similarly, EVs produced by ovarian carcinoma cells promoted the expression of α-SMA and FAP in normal ovarian primary fibroblasts [31]. In a rat model of PC, EVs derived from highly metastatic PC cells induced the expression of several wound healing genes, growth factors, chemokines and cytokines in primary prostate fibroblasts [32]. However, most of the previous studies have investigated the effects of EVs released by cancer cell lines. Here, we for the first time, studied the impact of urinary EVs on the transcriptional landscape of fibroblasts. A substantial fraction of urinary EVs has been shown to be derived from prostate or prostate cancer in PC patients [33]. Thus, we reasoned that urinary EVs from PC patients may be exploited for the analysis of functional effects of patients’ tumor-derived EVs.

The fibroblast cultures differed significantly in their response to the EV signaling. Cancer-naïve foreskin fibroblasts Hs68 had the highest number of genes regulated by EVs. However, the major part of genes regulated by PC- EVs was also regulated by HC-EVs and no unique pathways induced by PC-EVs were identified. At the same time, both PC-associated fibroblast primary cultures responded differently to PC-EVs and HC-EVs. One of the possible explanations is that dermal fibroblasts do not interact with PC-derived EVs but the effects are caused by other urinary EV subpopulations that are common between men with PC and healthy males. Alternatively, it may be possible that PC-derived EVs are not capable of triggering specific intracellular signaling pathways even if taken up or bound to dermal fibroblasts.

The transcriptional response of PCF-54 cells to PC-EVs differed substantially from that to HC-EVs. Treatment of PCF-54 cells with HC-EVs resulted in the downregulation of multiple mitochondrially encoded NADH dehydrogenase subunits and ATP synthase 6 and 8, as well as PDK4 encoding pyruvate dehydrogenase kinase 4 thus suggesting that the EV signaling may suppress the mitochondrial respiration. However, this process is likely to be dynamic and time-dependent, hence, it may be possible that the decrease that we observe at 48 h after adding EVs to the cell culture, in fact, follows an initial increase in the mitochondrial activity within the first hours of treatment. Interestingly, alterations in the mitochondrial respiration, including increased basal respiration, maximal respiration, and ATP-coupled respiration, have been also found in cytotoxic T cells treated with EVs derived from a melanoma cell line [34]. This suggests that the regulation of mitochondrial activity is a common effect elicited by EVs in various recipient cells. However, what is the molecular mechanism behind this effect and what are the characteristics of the EV subpopulation capable of eliciting this effect remains to be investigated.

The treatment of PCF-54 cells with PC-EVs affected the levels of 58 genes that were not regulated by HC-EVs. The genes upregulated by PC-EVs encode hepatocyte growth factor receptor MET, intracellular signaling molecules such as KRAS, RB1 and cyclin B2, helicases BRIP2 and HELLS and a number of proteins involved in the formation of the mitotic spindle, centrosomes and kinetochore, thus suggesting that PC-EVs interfere with the regulation of the cell division and chromosome segregation in CAFs.

In PCF-55 cells, both, PC-EVs and HC-EVs induced the expression of a number of chemokines such as CCL2, CCL13, CXCL1, CXCL8, etc. This finding is consistent with a previous report showing that EVs released by metastatic gastric cancer cells contribute to the generation of a specific subpopulation of CAFs producing CXCL family chemokines [14]. CCL2 binds to CCR2 and acts as a chemoattractant for monocytes and basophils [35, 36], whereas CCL13 binds to several receptors - CCR1, CCR2 and CCR3 - and functions as a chemoattractant for various immune cells, including eosinophils, basophils, monocytes, immature dendritic cells, and T cells [37]. CXCL1 is the activator and chemoattractant of neutrophils and it binds to glycosaminoglycans and CXCR2 receptors [38]. CXCL8 or IL-8 binds to CXCR1 and CXCR2 and attracts neutrophils, basophils and T cells, and it is involved in neutrophil activation and recruitment of MDSC to the TME [39]. These chemokines have been shown to promote angiogenesis, migration, epithelial-mesenchymal transition of PC cells as well as homing and establishment of metastasis [40, 41]. However, it has to be emphasized that this effect is not specific to PC-EVs but was also elicited by urinary EVs from healthy males.

Hence, our data support the idea that EVs contribute to the generation of functionally diverse fibroblast subpopulations [14]. It would be of great interest to investigate further the effects of various EV subpopulations on normal and activated prostate fibroblasts at a single cell level that may lead to deeper understating into the factors that determine the ability of fibroblasts to respond to the EV signaling and the functional consequences in terms of their contribution to recruiting various immune cells to the TME, development, and propagation of drug resistance, etc.

The EV uptake experiments showed that the majority of EVs are internalized into fibroblasts, while a fraction of EVs stayed attached to the cell membrane. However, what is the intracellular fate of EVs and how EVs trigger the alterations in the transcriptomes of recipient cells remains unknown. EVs carry a variety of proteins, lipids, carbohydrates, coding, and non-coding RNAs, DNA fragments, metabolites, and even entire organelles [5, 42–47]. Although EVs may release their RNA cargo in the recipient cells, we find it highly unlikely that it would directly alter the cellular RNA levels. More likely, the transcriptional changes are triggered by the activation of various signal transduction pathways by EV-enclosed proteins, miRNAs, or other signaling molecules or by binding of EVs to the cell surface receptors as it has been shown for T cells [10]. However, what is the contribution of different signaling molecules in changing the transcriptional landscape remains to be investigated.

The main limitation of our study is the small number of EV samples analyzed that precluded the identification of a gene expression signature commonly induced in fibroblasts by urinary EVs from PC patients. Nevertheless, this pilot study demonstrated that some fibroblast populations respond very differently to the urinary EVs from PC patients and healthy controls. This, in turn, suggests that EV recipient cells may be exploited as biosensors for detecting the presence of cancer. We believe that studying the transcriptional response of fibroblasts or other recipient cell types to EVs from a large cohort of patients and controls may lead to the identification of gene expression signatures that not only reveal the presence of cancer but also reflect its aggressiveness.

## Conclusion

To the best of our knowledge, this is the first study investigating the effects of urinary EVs from PC patients and healthy males on the transcriptional landscape of prostate CAFs and normal foreskin fibroblasts. Results show that the fibroblast lines significantly differ in their transcriptional response to the EVs thus supporting the idea that EVs contribute to the generation of functional heterogeneity of CAFs. Urinary EVs from PC patients and healthy males elicited distinct transcriptional responses in prostate CAFs suggesting that the changes in the gene expression pattern in EV recipient cells might serve as a novel type of functional cancer biomarkers.

## Electronic supplementary material

Below is the link to the electronic supplementary material.

Supplementary Material 1

Supplementary Material 2

Supplementary Material 3

Supplementary Material 4

## Data Availability

The RNAseq datasets are submitted in ArrayExpress: http://www.ebi.ac.uk/arrayexpress/experiments/E-MTAB-11022. For reviewer access: Username: Reviewer_E-MTAB-11,022. Password: gqqxfrE3.

## Declarations

The patients were enrolled in the study after the patients’ informed written consent was obtained.

The study protocol was approved by the Latvian Central Medical Ethics Committee (decision No. 01-29.1/488).

## Abbreviations

CAFs: Cancer-associated fibroblasts;
DEG: Differentially expressed gene;
EVs: Extracellular vesicles;
GO: Gene ontology;
NTA: Nanoparticle tracking analysis;
PC: Prostate cancer;
TEM: Transmission electron microscopy;
TME: Tumor microenvironment.
