# Supplementary figures and images for: Effects of urinary extracellular vesicles from prostate cancer patients on the transcriptomes of cancer-associated and normal fibroblasts
Source: BMC Cancer. 2022 Oct 12;22:1055. doi: 10.1186/s12885-022-10107-3 (PMC9555094; doi:10.1186/s12885-022-10107-3)

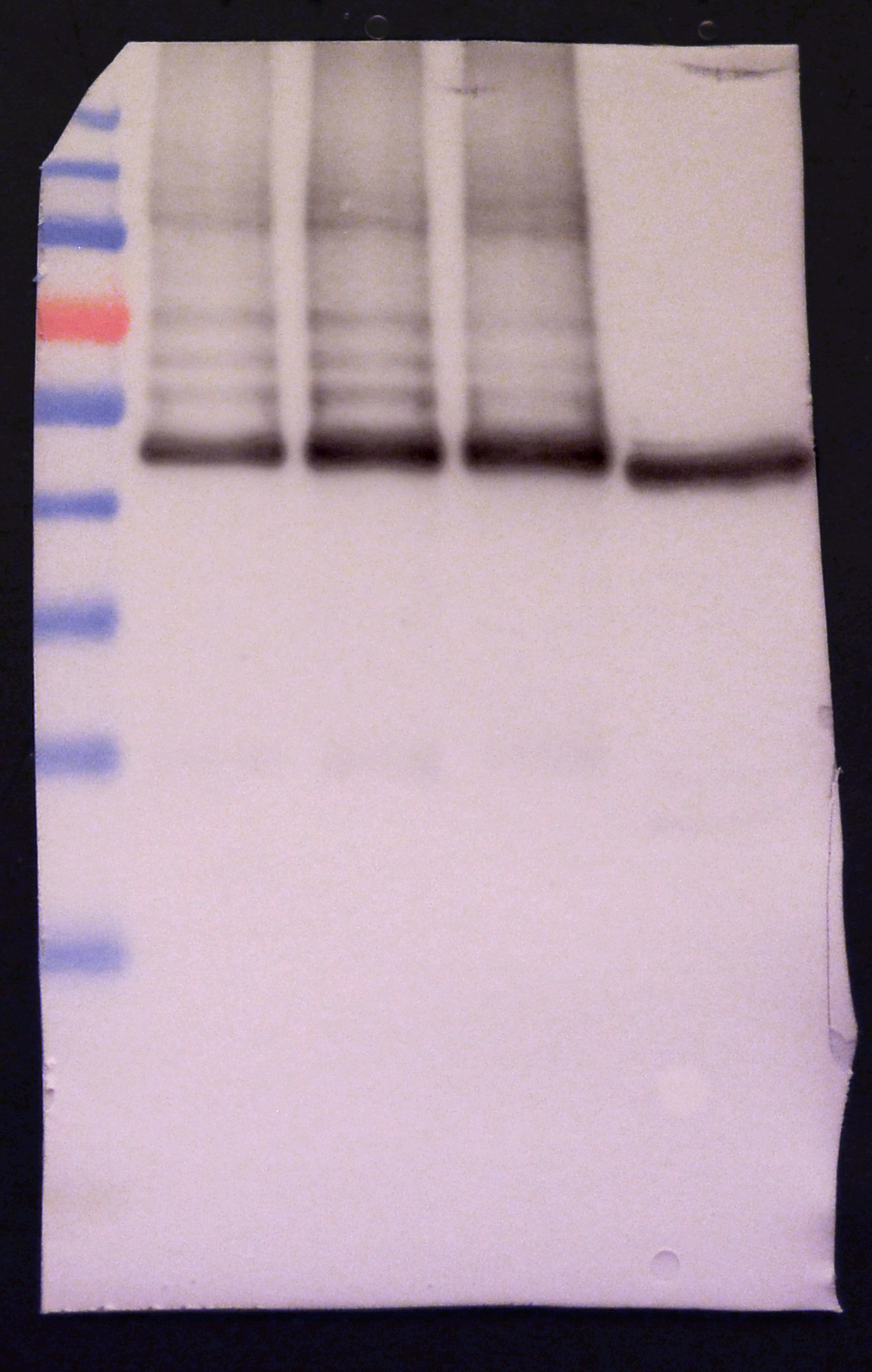

Supplement: Supplementary file 1 — Supplementary Material 1 [file 12885_2022_10107_MOESM1_ESM.jpg]

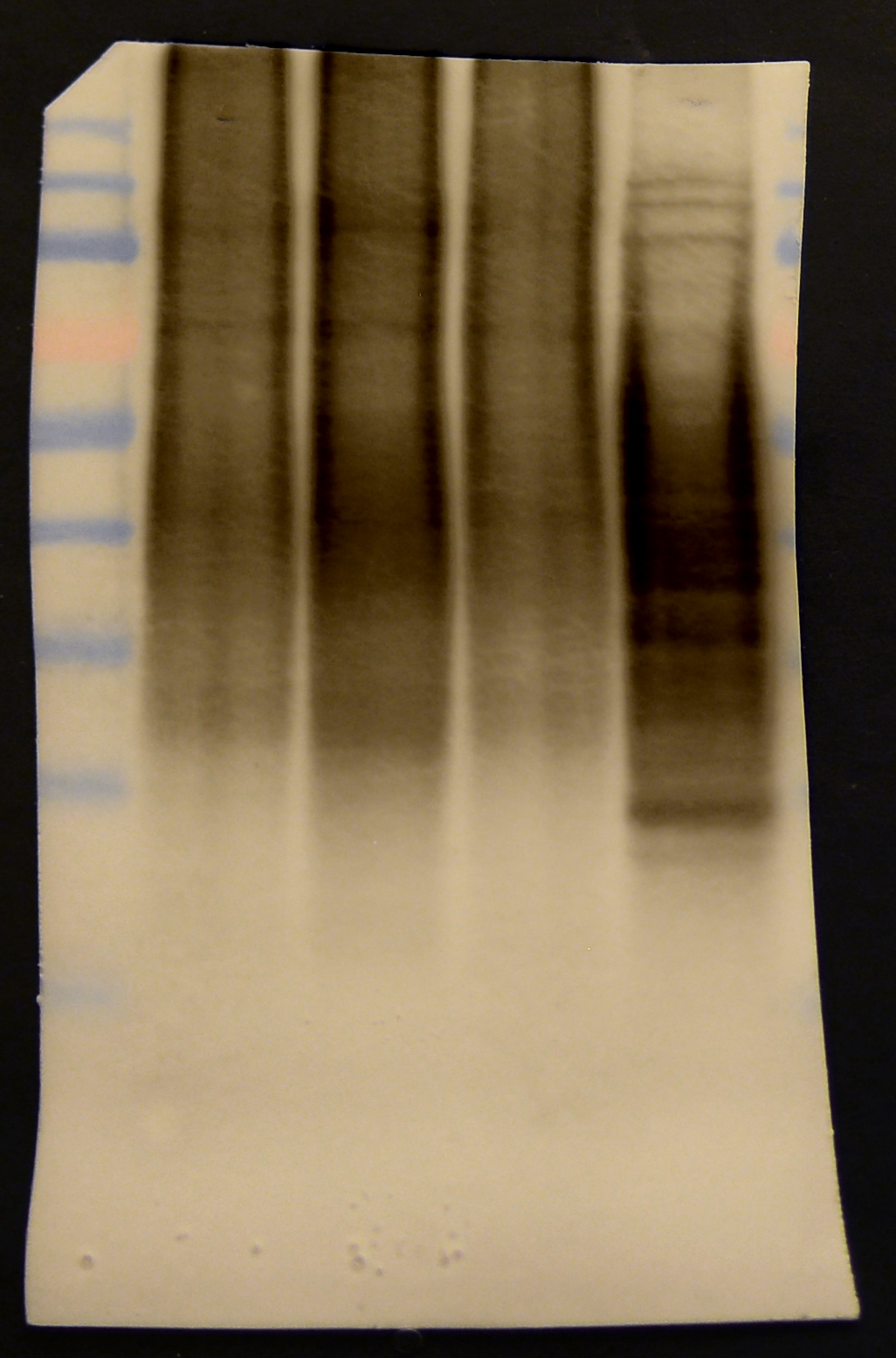

Supplement: Supplementary file 2 — Supplementary Material 2 [file 12885_2022_10107_MOESM2_ESM.jpg]

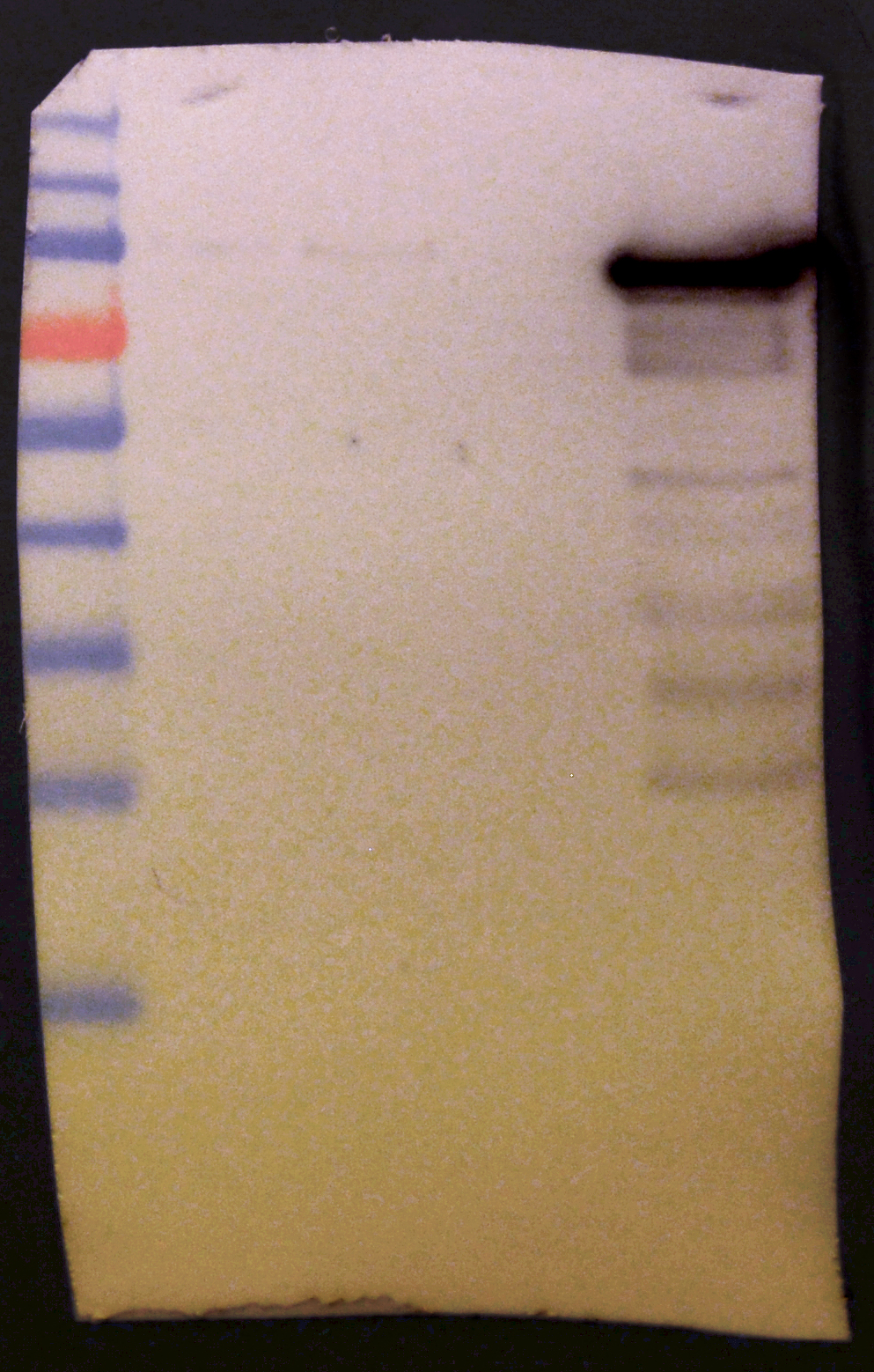

Supplement: Supplementary file 3 — Supplementary Material 3 [file 12885_2022_10107_MOESM3_ESM.jpg]
